# Quantification of Adaptive Immune Responses Against Protein-Binding Interfaces in the Streptococcal M1 Protein

**DOI:** 10.1016/j.mcpro.2024.100753

**Published:** 2024-03-23

**Authors:** Eva Torres-Sangiao, Lotta Happonen, Morizt Heusel, Frida Palm, Carlos Gueto-Tettay, Lars Malmström, Onna Shannon, Johan Malmström

**Affiliations:** 1 Faculty of Medicine, Division of Infection Medicine, Department of Clinical Sciences, Lund University, Lund, Sweden; 2 *Escherichia coli* Group, Health Research Institute of Santiago de Compostela (IDIS), Santiago de Compostela, Spain; 3 Clinical Microbiology Lab, University Hospital Complex of Santiago de Compostela, Santiago de Compostela, Spain; 4 Evosep ApS, Odense, Denmark; 5 Faculty of Odontology, Section for Oral Biology and Pathology, Malmö University, Malmö, Sweden

**Keywords:** *Streptococcus pyogenes*, SWATH-MS, immune response, mouse model, host-pathogen interactions

## Abstract

Bacterial or viral antigens can contain subdominant protein regions that elicit weak antibody responses upon vaccination or infection although there is accumulating evidence that antibody responses against subdominant regions can enhance the protective immune response. One proposed mechanism for subdominant protein regions is the binding of host proteins that prevent antibody production against epitopes hidden within the protein binding interfaces. Here, we used affinity purification combined with quantitative mass spectrometry (AP-MS) to examine the level of competition between antigen-specific antibodies and host–pathogen protein interaction networks using the M1 protein from *Streptococcus pyogenes* as a model system. As most humans have circulating antibodies against the M1 protein, we first used AP-MS to show that the M1 protein interspecies protein network formed with human plasma proteins is largely conserved in naïve mice. Immunizing mice with the M1 protein generated a time-dependent increase of anti-M1 antibodies. AP-MS analysis comparing the composition of the M1-plasma protein network from naïve and immunized mice showed significant enrichment of 292 IgG peptides associated with 56 IgG chains in the immune mice. Despite the significant increase of bound IgGs, the levels of interacting plasma proteins were not significantly reduced in the immune mice. The results indicate that the antigen-specific polyclonal IgG against the M1 protein primarily targets epitopes outside the other plasma protein binding interfaces. In conclusion, this study demonstrates that AP-MS is a promising strategy to determine the relationship between antigen-specific antibodies and host-pathogen interaction networks that could be used to define subdominant protein regions of relevance for vaccine development.

## Highlights

• Strategy to determine the relationship between antigen-specific antibodies and host-pathogen interaction networks.
• Host-pathogen interaction networks formed with human plasma proteins conserved in naïve mice.
• Novel animal model to study strategies to tackle *Streptococcus pyogenes* infection.

## Graphical Abstract

**Figure:**
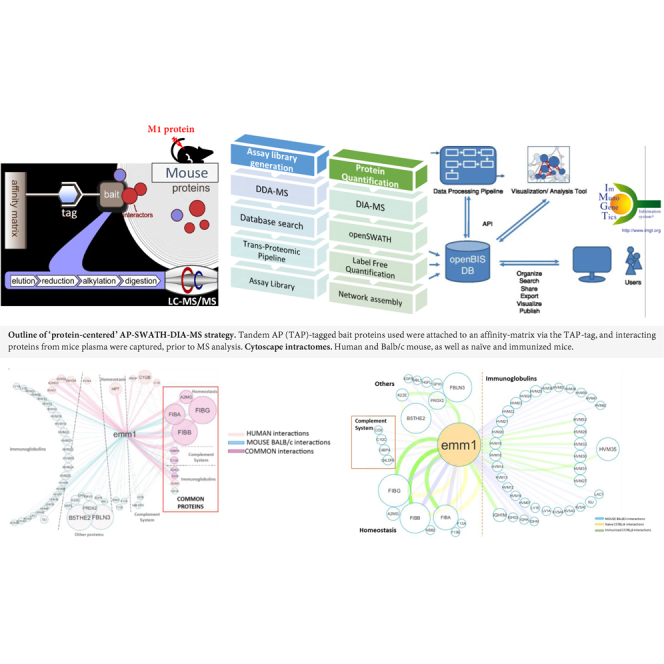

## In Brief

Here, we used affinity-purification combined with quantitative mass spectrometry to examine the level of competition between antigen-specific antibodies and host-pathogen protein interaction networks using the M1 protein from *Streptococcus pyogenes* as a model system. The results indicate that the antigen-specific polyclonal IgG against the M1 protein primarily targets epitopes outside the other plasma protein-binding interfaces.

Most licensed vaccines confer protection against infectious diseases by eliciting an adaptive immune response to generate pathogen-specific antibodies (1). These adaptive immune responses are efficient in many cases but protection is often limited to the infecting or immunizing strain (2). Recent findings suggest that immunodominance of antibody responses is a limiting factor that may be of particular interest for vaccine development (2, 3). Immunodominance is the hierarchical focusing of adaptive immune responses to a subset of antigenic determinants (2) and signifies that one part of an antigen elicits a stronger antibody response than another comparable part of the antigen. A recurring problem observed for several pathogens seems to be that the production of antibodies to immunodominant antigenic sites sometimes elicits poor functional antibody responses (3, 4). In contrast, there is accumulating evidence that protective antibodies can be induced against crucial subdominant components of a pathogen that in natural infection induces only a weak antibody response (4, 5, 6). Consequently, understanding the molecular mechanisms behind immunodominance and subdominance is important to understand pathogen evasion of antibody responses and design vaccines that elicit antibodies against protective, evolutionarily conserved antigenic sites (2).

A mechanism that contributes to subdominance is the binding of host proteins to proteins from pathogens (7, 8). Binding of host proteins can subvert antibody production against epitopes found in the protein binding interfaces (7, 8, 9). It is well known that many bacteria have evolved several mechanisms by which they can acquire host proteins on their surface (10) that play key roles in pathogenesis (11). Recent advances in methods such as affinity-purification mass spectrometry (AP-MS) have generated novel information regarding protein-protein interactions between bacterial proteins (12, 13, 14) and between host proteins and pathogens (15). Such information has paved the way for system-level analyses of whole interactomes, leading to a better understanding of pathogenicity, disease progression, and human-pathogen coevolution (16). Further, the development of chemical crosslinking mass spectrometry (XL-MS) (17, 18) and hydrogen-deuterium exchange mass spectrometry (HDX-MS) (19, 20) has increased the resolution of these interactions by catalyzing the identification of protein-binding interfaces, which is of relevance for the uncovering of systems-level molecular recognition principles governing host-bacteria interactions (21). In addition, the accumulation of host-pathogen protein-protein interaction interfaces provides new opportunities to investigate the relationship between antibody responses and protein binding interfaces.

*Streptococcus pyogenes* (or Group A *streptococcus*, GAS) is a Gram-positive bacterium and a significant human pathogen that forms extensive protein interaction networks with human host proteins. Different microenvironments alter the composition of the interaction networks depending on the protein composition found in different ecological niches (15, 22). Infections with *S. pyogenes* range from relatively mild superficial throat and skin infections to severe and life-threatening conditions such as necrotizing fasciitis and streptococcal toxic shock syndrome (23). One of the main virulence factors produced by *S. pyogenes* is the M protein (24) that harbors several protein-binding domains that mediate binding to human albumin, fibrinogen, and the fragment crystallizable (Fc)-domain of IgG molecules (25, 26, 27). The N-terminal 50 amino acid residues constitute the hypervariable region (HVR), which is used to classify clinical isolates of *S. pyogenes* into M (*emm*) types (28). A globally prevalent serotype in severe streptococcal disease is *emm*1. In the M1 protein, the HVR is followed by a stretch of 100 to 150 amino acids that forms the semi-variable domain and encompasses the A domain and the B repeats. The subsequent C repeats and the D domain form the conserved C-terminal part of the M1 proteins (29, 30). Recent XL-MS analysis and structural modeling identified host-pathogen protein binding interfaces within all domains of the M1 protein. These protein binding interfaces interact with up to ten different human proteins to potentially form a large 1.8-MDa interspecies protein complex (18, 31, 32). Previous reports have shown that antibodies elicited by the M proteins from both mice and humans are mostly directed against the immunodominant C-terminal region, whereas the B repeats and the HVR regions were proposed to be inherently weekly immunogenic (33, 34). These findings raise the question of whether protein binding interfaces between the M1 protein and other human plasma proteins influence the location of dominant and subdominant protein regions. Due to the high frequency of streptococcal infections, most humans have been exposed to *S. pyogenes* and have circulating antibodies against streptococcal antigens (35) making it challenging to find completely naïve human subjects. Another complicating factor is that the alpha-helical coiled-coil structure of M protein resembles the structure of host proteins such as myosin, keratin, tropomyosin, vimentin, and laminin that can lead to cross-reactive autoantibodies (36). The presence of an existing repertoire of circulating antibodies complicates investigations focusing on the interplay between polyclonal antibody mixtures and host-pathogen protein-protein interactions.

Here, we addressed this challenge by immunizing naive mice with M1 protein. We demonstrate that the M1 protein forms an interspecies protein interaction network with plasma proteins from mice that resembles the protein interaction network formed with human plasma proteins. Immunization with the M1 protein in mice elicited strong anti-M1 titers but competition experiments revealed that these specific antibodies could only to a limited extent prevent the protein interactions formed with the mouse plasma proteins. These results indicate that the anti-M1 antibodies post-immunization are directed to epitopes outside the protein binding interfaces and that the protein-binding interfaces could represent subdominant regions.

## Experimental Procedures

### Cloning, Protein Expression, and Purification

The *Streptococcus pyogenes* open reading frames (ORFs) (amino acids 42–484) encoding for the protein M1 (UniProt ID: Q99XV0, emm1), as well as super folder green fluorescent protein (sfGFP) used as a negative control in our AP-MS experiments, were cloned, expressed and purified at the Lund Protein Production Platform (LP3) as described (37). The encoding sequence was ordered as a synthetic construct from Genscript, cloned into the EcoRV site of pUC57, and subsequently subcloned into a pNIC28-Bsa4-based vector carrying the tandem affinity purification (TAP) tag used in this study (6× His-HA-StrepII-TEV [histidine-hemagglutinin-StrepII-tobacco etch virus protease recognition site]) at the C-terminus of the construct.

The protein M1 was expressed in Luria-Bertani Broth (LB) (Difco) at 37 °C in *Escherichia coli* BL21 (DE3) cells. Protein expression was induced with 1 mM IPTG at OD_600_ 0.5 to 0.6. Protein M1 was purified from harvested cells using a fibrinogen column as described (37). Pooled fractions from the fibrinogen column were dialyzed against 1 × PBS pH 7.4 and loaded on Ni-coupled Imac Sepharose 6 Fast Flow (GE Healthcare). The column was washed with 20 mM imidazole in 1 × PBS pH 7.4, and bound protein was eluted with 500 mM imidazole in 1 × PBS pH 7.4 using gravity flow. Pooled fractions from the Ni-column were buffer exchanged into 1 × PBS pH 7.4 and concentrated using Millipore Amicon 10,000 kDa or 30,000 kDa molecular weight cutoff concentrators. Purified protein was stored at −80 °C until usage.

The overall quality and purity of all proteins used in this study were analyzed *via* SDS-PAGE and LC-MS/MS of after tryptic digest. All the proteins used in this study are presented in Happonen L., *et al.* (15).

### Commercial Proteins, Human, and Mouse Plasma

Pooled commercial human and BALB/c mouse plasma were purchased from Innovative Research. An aliquot of each sample, human and mice plasmas, as well as the mixed human-mouse, were diluted at 1:1000 and prepared for MS as described below. Citrate was used as an anticoagulant.

### Immunization With M1 Protein

The animal use protocol was approved by the local Malmö/Lund Institutional Animal Care and Use Committee (M115-13). The initial experiments were performed commercially available and purchased BALB/c plasma. For immunization, we switched to C57/BL6J mice since this is our strain of choice for immunization and infection models with *S. pyogenes*. C57BL/6J is SPF (Specific Pathogen free). The mice are purchased from an SPF facility (Janvier) and then housed in an SPF facility in Lund in cages with isolated airflow in and out of each cage. We expect no interference from previous colonization. Nine-week-old female C57BL/6J mice (Janvier) were injected subcutaneously with M1 protein on days 1, and 21 (10 μg/dose combined 50:50 with Titermax Gold adjuvant). Control mice were injected with adjuvant alone. After 6 weeks all mice were sacrificed, and citrated plasma was collected by cardiac puncture. Anti-M1antibody titters by were determined using an in-house ELISA as previously described (38).

### Affinity-Purification in Human and Mouse Plasma

The affinity-purification reactions using both human and mouse plasma were done essentially as described (39). Briefly, Strep-Tactin Sepharose beads (IBA) were packaged in a Bio-Spin Chromatography Columns (BioRad) and equilibrated in PBS buffer, pH 7.4, for afterwards charged with 10 μg of recombinant TAP-tagged bait, M1 and sfGFP, proteins. TAP-tagged sfGFP was used as a negative control in all experiments. Pooled human, BALB/c mouse or C57BL/6 mouse, plasma was incubated with the protein-charged beads at 37 °C, 800 rpm, 1 h. The beads were washed with 7 ml of ice-cold PBS buffer, pH 7.4, at 4 °C, and the proteins were eluted using 120 μl of 5 mm biotin in PBS buffer, pH 7.4 at room temperature (39). The samples were precipitated with tri-chloro acetic (TCA), and finally reduced, alkylated and trypsin digested for mass spectrometry.

### Bacterial Strains and Culture Conditions

The *S. pyogenes* strains SF370, a clinical isolate of the M1 serotype; and its isogenic M1-mutant (ΔM1) were used for adsorption experiments (39, 40). The bacteria were grown on blood agar plates and or from single colonies in Todd-Hewitt broth supplemented with 0.3% (w/v) yeast extract (THY-media) (18). The bacteria were grown at 37 °C, 5% CO2 in until mid-logarithmic phase (OD620 nm 0.4–0.5), harvested by centrifugation (3500*g*, 5 min, RT), and the cell pellets were washed with a total of 3 ml HEPES buffer (50 mM HEPES, 150 mM NaCl pH 7.5). The cells were re-dissolved in HEPES to a concentration of 1% bacterial solution (approximately 700 μl per 10 ml of original culture) and used for plasma adsorption experiments as described below.

### Plasma Adsorption in Pooled BALB/C Mouse Plasma and Surface Shave

The plasma adsorption protocol has been described (25, 41, 42). Briefly, 100 μl of the bacteria solution was mixed with 400 μl of BALB/c mouse plasma, and incubated at 37 °C, 500 rpm, 30 min. The bacteria with adhered plasma proteins were harvested by centrifugation (500*g*, 5 min, RT) and washed with a total of 1.5 ml HEPES buffer. The pellets were resuspended in 100 μl HEPES. The surface proteins with attached plasma proteins were digested off with 2 μG trypsin (Promega) at 37 °C, 500 rpm, 1 h, as previously described. The reaction was stopped, and bacteria were harvested by centrifugation (1000*g*, 15 min, 4 °C), supernatant recovery, and heat inactivation of any remaining pathogenic bacteria (85 °C, 5 min). The cells were finally reduced, alkylated, and digested for mass spectrometry.

### Sample Preparation for MS

The samples from AP and PA experiments were mixed with 8 M urea and ammonium bicarbonate (ABC), and the cysteine bonds were reduced with 5 mM *tris*(2-carboxyethyl) phosphine (TCEP) (37 °C, 30 min) and alkylated with 5 mM iodoacetamide (IAA) (dark, RT, 1 h). Samples were diluted with ABC to a final urea concentration of 1.5 M, and sequencing-grade trypsin (Promega) was added for protein digestion (37 °C for 18 h). Samples were acidified with 10% formic acid to a final pH of 3.0, and the peptides subsequently purified with SOLAμ reverse phase solid phase extraction plates according to the manufacturer's instructions (Thermo Fisher). Peptides were dried in a speed-vac and reconstituted in 2% acetonitrile, 0.1% formic acid prior to mass spectrometric analyses. To mitigate variation, we used next biological replicates per experiment: 9 biological replicates for Human plasma, 7 for human-mouse competition and Balb/c or C57BL/6 naive/immunized affinity purification experiments, 6 biological replicates for plasma adsorption experiments, and 3 biological replicates for plasma samples.

### Liquid Chromatography-MS

#### Data-Dependent Acquisition

For AP data-dependent acquisition (DDA), all peptides were performed on Q Exactive Plus mass spectrometer (Thermo Scientific) connected to an EASY-nLC 1000 ultra-high-performance liquid chromatography system (Thermo Scientific) as has been described (43). Peptides were separated on an EASY-Spray column (Thermo Scientific; ID 75 μm 25 cm, column temperature 45 °C) operated at a constant pressure of 600 bar. A linear gradient from 5 to 35% acetonitrile in aqueous 0.1% formic acid was run for 120 min at a flow rate of 300 nl min^−1^. One full MS scan (resolution 70,000 @ 200 m/z; mass range 400–1 600 m/z) was followed by MS/MS scans (resolution 17,500 @ 200 m/z) of the 15 most abundant ion signals. The precursor ions were isolated with 2 m/z isolation width and fragmented using higher-energy collisional-induced dissociation (HCD) at a normalized collision energy of 30. Charge state screening was enabled, and precursors with an unknown charge state and singly charged ions were rejected. The dynamic exclusion window was set to 10 s. The automatic gain control was set to 1e6 for both MS and MS/MS with ion accumulation times of 100 ms and 60 ms, respectively. The intensity threshold for precursor ion selection was set to 1.7e4.

For PA-DDA, all peptides were performed on Q Exactive HF-X mass spectrometer (Thermo Scientific) connected to an EASY-nLC 1200 ultra-high-performance liquid chromatography system (Thermo Scientific) as has been described (43). Peptides were separated on an EASY-Spray column (Thermo Scientific; ID 50 μm 25 cm, column temperature 45 °C) operated at a constant pressure of 800 bar. A linear gradient from 4 to 45%, 80% acetonitrile in aqueous 0.1% formic acid was run for 65 min at a flow rate of 350 nl min^−1^. One full MS scan (resolution 60,000 @ 200 m/z; mass range 390–1 210 m/z) was followed by MS/MS scans (resolution 15,000 @ 200 m/z) of the 15 most abundant ion signals. The precursor ions were isolated with 2 m/z isolation width and fragmented using HCD at a normalized collision energy of 30. Charge state screening was enabled, and precursors with an unknown charge state and a charge state of 1, 6 to 8, >8 were rejected. The dynamic exclusion window was set to 10 s. The automatic gain control was set to 3e6 and 1e5 for MS and MS/MS with ion accumulation times of 110 ms and 60 ms, respectively. The intensity threshold for precursor ion selection was set to 1.7e4.

#### Data-Independent Acquisition MS

All peptide analyses were performed on a Q Exactive HF-X mass spectrometer (Thermo Scientific) connected to an EASY-nLC 1200 ultra-high-performance liquid chromatography system (Thermo Scientific) as has been described (43). Peptides were separated using an EASY-Spray column (Thermo Scientific; ID 50 μm × 25 cm, column temperature 45 °C) operated at a constant pressure of 800 bars. For AP data-independent acquisition (DIA) a linear gradient from 3% to 45% of 80% acetonitrile in aqueous 0.1% formic acid was run for 65 min at a flow rate of 350 nl min^−1^. A full MS scan (resolution 120,000 @200 m/z; mass range from 350 to 1650 m/z) was followed by 24 MS/MS full fragmentation scans, for AP experiments, (resolution 30,000 @ 200 m/z) using a variable isolation window. The precursor ions within each isolation window were fragmented using HCD at a normalized collision energy of 27. The automatic gain control was set to 3e6 for both MS and MS/MS with ion accumulation times of 100 ms and 50 ms, respectively. For SB-DIA a linear gradient from 4% to 45% of 80% acetonitrile in aqueous 0.1% formic acid was run for 65 min at a flow rate of 350 nl min^−1^. A full MS scan (resolution 60,000 @200 m/z; mass range from 390 to 1210 m/z) was followed by 32 MS/MS full fragmentation scans (resolution 30,000 @ 200 m/z) using a fixed isolation window set at 26 m/z. The precursor ions within each isolation window were fragmented using HCD at a normalized collision energy of 30. The automatic gain control was set to 1e6 and 3e6 for MS and MS/MS with ion accumulation times of 120 ms and 100 ms, respectively.

### MS Data Analysis

MS raw data were converted to gzipped and Numpressed (44) mzML using the tool MSconvert from the ProteoWizard, v3.0.5930 suite (45). All data analyses were stored and managed using openBIS (46). MS searches were performed as described (25, 47, 48).

The human spectral library was generated from the PeptideProphet data using the Fraggle-Franklin-Tramler pipeline (49) against an in-house compiled database containing the *Homo sapiens* references proteome with the *S. pyogenes* protein M added. The mouse spectral library used for SWATH quantification was created according to the workflows included in openBIS (46), including the sequence for streptococcal M1 protein.

MS raw data were converted to gzipped and Numpressed (44) mzML using the tool MSconvert from the ProteoWizard, v3.0.5930 suite (45). All data analyses were stored and managed using openBIS (46). MS searches were performed as described (25, 47, 48). Briefly, DDA acquired spectra were analyzed using the search engine X! Tandem (2013.06.15.1-LabKey, Insilicos, ISB) (50) and OMSSA (51) towards a human or mouse reference database with reverse decoys, follow by a *Trans*-Proteomic Pipeline (TPP v4.7 POLAR VORTEX rev 0, Build 201,403,121,010) using PeptideProphet (52). The false discovery rate (FDR) was estimated with Mayu (v1.7) and peptide spectrum matches (PSMs) were filtered with protein FDR set to 1% resulting in a peptide FDR >1%. Nonredundant consensus spectral libraries were built with TPP Spectrast using the filtered PSMs with all retention times converted to iRTs (53) and selecting the top six most intensive transitions per assay (tier level 3 analysis).

Human UniProt proteome IDs UP000005640 with 72,241 protein entries, *S. pyogenes* UniProt UP000000750 with 22,262 protein entries, and mouse EMBL-EBI release 2018_04 UP000000589 10,090 MOUSE *Mus musculus* with 55,021 protein entries were used to generate the respectively spectral libraries with or without *S. pyogenes* protein M1 added. The human spectral library was generated from the PeptideProphet data using the Fraggle-Franklin-Tramler pipeline (49). Fully tryptic digestion was used allowing 1 missed cleavage. Carbamidomethylation (C) was set to static, and oxidation (M) was set to variable modifications, respectively. The mass tolerance for precursor ions was set to 0.2 Da, and that for fragment ions was set to 0.02 Da. The mouse spectral library used for SWATH quantification was created according to the workflows included in openBIS (46). Briefly, target assays were generated using spectraST, FDR calculations of 1% for peptide and protein were calculated with CLI, and feature alignment with TRIC using the following settings: mass extraction window = 0.05 Da, RT-extraction window = 300 s and Max RT-difference of 30, requiring a minimum of 4 matched fragment peaks (54). Acquired spectra were analyzed against an in-house compiled database containing the *M. musculus* and *S. pyogenes* with/without protein M added.

### Immunoglobulin Data Analysis

To retrieve additional metadata for the immunoglobulins found enriched on M1 bait protein in immunized animals compared to control animals, we retrieved the full sequences for the identified Immunoglobulins from UniProt. These sequences were then searched against the IMGT immunoglobulin database (https://www.imgt.org/) using tblastn with default settings for (gap opening penalty = 11, Gap extension penalty =1, Expectation value = 10.0 and the blosum62 as substitution matrix for amino acid comparisons). The top hit accession was selected to then retrieve further information *via* search using the IMGT/LIGM-DB query form with standard parameters and selecting “Mouse” as species and “IgG” as receptor type (http://www.imgt.org/ligmdb/search.action). Finally, results from IMGT-V-Quest module were exported in Excel format.

### Experimental Design and Statistical Analyses

Data was analyzed using Jupyter Notebooks (version 3.1.1, 2014-07-10) and Adobe Illustrator CS5 (Adobe Systems). Statistical significance between sample replicates was evaluated with a 2-sided student *t* test where a *p*-value of <0.01 was considered statistically significant. The SWATH data was filtered according to a *p*-value <0.01 and log2 fold enrichment ≥2, using a bait to GFP. For interactomes, all proteins passed the above selection criteria and were identified by ≥3 peptides. Additionally, the statistically significant proteins were identified by a *q*-value (Benjamin – Hochberg adjustment) <0.05.

## Results and Discussion

### The M1 Protein—Human and Mouse Interactome

**Fig. 1:**
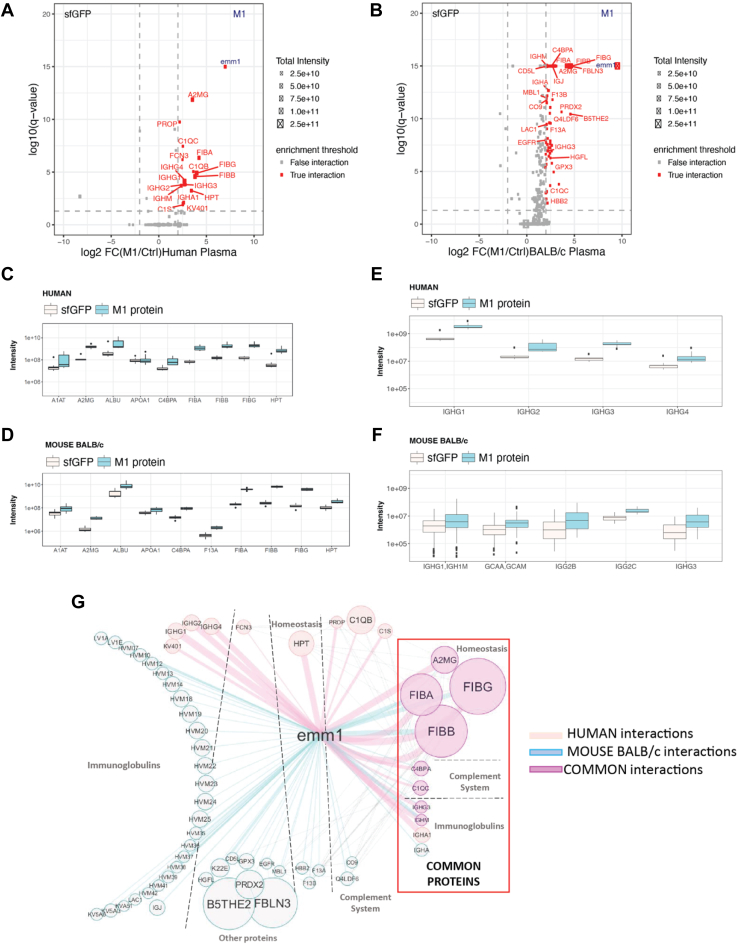
**M1 protein affinity-purification of human and mouse plasma proteins.***A* and *B*, differential abundance of mouse and human plasma proteins interacting with the M1 protein after AP-MS. The volcano plots display statistically significant enriched interactions after Benjamini-Hochberg adjusted *q*-value <0.05 on the y-axis and the fold changes (FC) between M1 protein and sGFP on the x-axis (M1/sfGFP). The dashed *grey* lines indicate the set thresholds corresponding to log10 (*q*-value <0.05) and log2 fold change ≥2. The intensity of the proteins is shown by size of the dots. Proteins that pass the filtering criteria are marked as high-confident or TRUE interactions (*red dots*) while *grey dots* indicate contaminating proteins or FALSE interactions falling below the set thresholds (57). The M1 protein is denoted as emm1 in *blue font*. *C* and *D*, boxplots of the intensity distribution for enriched plasma proteins after AP-MS using sfGFP (Ctrl) and M1 protein (M1) as bait in human plasma (*C*) and mouse plasma (*D*). The intensity for alpha-2-macroblobulin (A2MG) and mouse A1AT was calculated as mean of the six domains (A1AT*1-6*). See supplemental Table S1 for more information and protein acronyms. *E* and *F*, boxplots of the IgG subclass distribution after AP-MS using sfGFP (Ctrl) and M1 protein (M1) as bait in human plasma (*E*) and mouse plasma (*F*). The intensities for the IgG subclasses were quantified based on specific and conserved proteotypic peptides in the different Fc-domain subclasses as previously described (18). Acronyms: emm1 = M1 protein of *S. pyogenes (emm1).* IGH1M = IGHG1 membrane; GCAA = A alele and GCAM = membrane are IGG2A. *G*, the M1 protein – human and BALB/c mouse plasma interactome of stringently filtered proteins. Known human-human and mouse-mouse interactions from the STRING-database are depicted using dashed *grey lines*. The line width is based on the log10 normalized mean protein intensity and node size is based on fold change between M1 protein and sfGFP samples. The interactome views were generated using Cytoscape and modified in Adobe Illustrator. The STRING interactions were generated by Cytoscape setting the confidence score at 0.9.

To determine if mice represent a suitable model system, we first investigated the level of conservation for the protein interactions formed between the M1 protein and human and mouse plasma proteins. To this end, we used a recombinantly expressed, affinity-tagged M1 protein to affinity-enrich pooled mouse or human plasma proteins followed by sequential DDA- and DIA-MS analysis. The identified proteins were filtered against identified proteins from superfolder green fluorescent protein (sfGFP) affinity-enriched samples as a negative control using a fold change of ≥2, an adjusted *p*-value of <0.05 (*q*-value) and requiring at least three unique peptides (Fig. 1, *A* and *B*). Based on these thresholds, the experiments generated in total of 17 interacting human plasma proteins and 50 interacting BALB/c mouse plasma proteins and 39 variable mouse immunoglobulin fragments although the number of Ig fragments was similar on both the murine and human FASTA file (Fig. 1, *A* and *B*). Compared to previously published results (23), we confirm several predominant M1-binding protein classes in the human plasma-M1 protein interactome, such as immunoglobulins, fibrinogens, albumin, haptoglobin, and members of the complement system such as the human complement system C4b-binding protein (C4BP) (Fig. 1, *A* and *C*). Several of the interactions such as albumin, fibrinogen, C4BP, and alpha-2-macroglobulin (A2MG) were also formed between the M1 protein and mouse plasma proteins (Fig. 1, *B* and *D*).

Previous work has shown that IgG3 is the predominant subclass for M protein-mediated fragment antigen-binding (Fab)-region-bound antibodies in humans, whereas human IgG1, 2, and 4 are partly bound *via* the Fc-domain to the M1 protein (15, 18). When focusing on tryptic peptides from conserved constant domains of subclass-distinct protein regions from the different subclasses of human IgGs, we confirm higher enrichment of IgG3 (>10-fold) in the M1 protein AP-MS data compared to IgG1, 2, and 4 (Fig. 1*E*). In contrast, the results from the mouse plasma analysis showed similar levels of mouse immunoglobulin IgGs comparing the M1 protein and sfGFP pulldowns mediated by Fc-domain binding to the M1 protein (Fig. 1*F*). Immune bound antibodies can further recruit binding of human complement system C1q complex to the streptococcal surface in immune plasma (15, 18). The interaction with C1q, C1S, C1R, and the M1 protein is dependent on the Fc-domain from Fab-bound antibodies, as previous work has shown that removal of the Fc-domain using IdeS completely abolishes affinity purification of C1q complex using the M1 protein (18). Here, we identified C1q enriched on the M1-protein only when using human plasma. It currently remains unclear if this binding is mediated by pre-existing circulating antibodies in human plasma derived from previous exposures or autoantibodies against structurally similar host proteins. In mouse plasma, however, we do not observe recruitment of the C1q complex to the same extent, which is to be expected since naïve mice do not have specific Fab-bound antibodies to the M-protein.

The identified protein interaction network formed between the M1 protein and human/mice plasma was subsequently visualized using an interactome map (Fig. 1*G*). The interactome map shows statistically enriched proteins as circles compared to the negative control, where the circle size is proportional to protein abundance. Colors indicate if a particular protein was identified only from human or mouse plasma or from both species. The interactome map shows that the three chains of fibrinogen are the most abundant interactions that were specifically affinity-enriched *via* M1 in both species, followed by A2MG, C4BP, and various immunoglobulins. Human-specific interactions were haptoglobin, ficolin, C1qB, C1s, and IgG1, 2 and 4. In contrast, the mouse affinity-purification experiments identified fibulin, maltase, glucoamylase, and coagulation factor 13A and 13B (F13A and F13B) as binders. Coagulation factor 13A was in previous reports shown to interact with the M1-complex *via* binding to fibrinogen (18). Here, human F13A was statistically enriched in the M1-purification experiments but was filtered out as it was only identified using two unique peptides, still indicating that F13A and fibrinogen binding on the M1 protein complex is conserved between the two species. In contrast, haptoglobin was identified as a significant interaction from the human AP-MS experiments but only enriched in the murine AP-MS experiments without reaching statistical significance. The human C4BP interacts with the HVR of many M proteins (55) *via* conserved sequence patterns hidden within hypervariable regions (38). This interaction is also formed in mouse plasma, which indicates that conserved structural domains between mouse and human versions of C4BP is responsible for the interaction; although the binding to the M1 protein is weaker compared to other M-proteins (22). In conclusion, these results demonstrate that the M1 protein interaction network formed with mouse plasma proteins is like the M1-human protein interaction network, except for IgG and C1q.

### Properties of the Human and Mouse Plasma M1 Protein Interactome

**Fig. 2:**
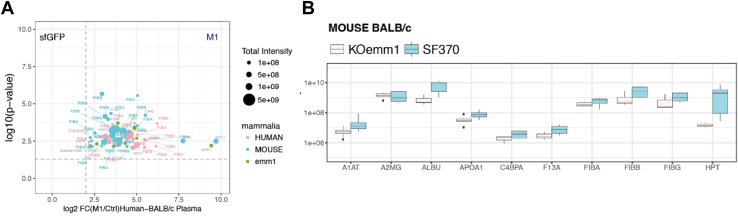
**Properties of the human and mouse plasma M1 protein interactome.***A*, a tagged M1 protein was used to enrich mouse and human plasma proteins from a (50% vol/vol) mixture of human and BALB/c mouse plasma followed by mass spectrometry analysis. The volcano plots display statistically significant enriched interactions after Benjamini-Hochberg adjusted *q*-value <0.05 on the y-axis and the fold changes (FC) between M1 protein and sGFP on the x-axis (M1/sfGFP). The *dashed grey-lines* indicate log10 (*q*-value <0.05) and log2 fold change ≥2. The intensity for each protein is shown by size of the dots. *B*, surface adsorption experiments using mouse plasma and SF370 as the bacterial strain and a ΔM1 protein isogenic mutant referred to as KO *emm1(ΔM1)*. The boxes show the intensity range for each protein in wtSF370 and the ΔM1 protein isogenic mutant KO *emm1* samples.

Although the overall composition of the M1-human/murine interaction network is similar the interaction affinities could be substantially different. To address this, we performed competition experiments using a mixture (50% vol/vol) of human and BALB/c mouse plasma to measure if the M1 protein predominately binds human proteins in equal amounts of human and murine plasma. The plasma mixture composed of both mouse and human plasma proteins was used to affinity-enrich interacting proteins using the M1 protein and sfGFP as baits followed by DIA-MS. The identified peptides were displayed in a peptide-centric view focusing on peptides that were unique to each species to distinguish between human and mouse proteins (Fig. 2*A* and supplemental Fig. S2). In this analysis, it is apparent that limited competition occurs since no significant differences were observed between the species-specific peptides. The reason could be that the binding between M1 and the plasma proteins occurs at structurally conserved binding interfaces.

To confirm that the mouse plasma and M1 interactions also occur at the bacterial surface, we performed surface-adsorption in mouse plasma as previously described (18), using both a wild-type *S. pyogenes* strain and an isogenic ΔM1 mutant as negative control. In these experiments, the wildtype SF370 strain significantly enriched several proteins compared to the mutant ΔM1 after filtering interactions based on an adjusted *p*-value of ≤0.05 (*q*-value). Compared to the ΔM1 strain, the wildtype strain enriched several of the known M1 protein binders such as the three chains of fibrinogen, albumin, and haptoglobin more than 10-fold (Fig. 2*B*).

In conclusion, these results demonstrate that the interactions between the M1 protein and mouse plasma proteins occur on the bacterial surface. In addition, the competition experiments demonstrate that the binding of mouse plasma proteins is not significantly diminished by the presence of human plasma proteins.

### M1 Protein—Mouse Plasma Complex in Plasma from Immunized Mice

**Fig. 3:**
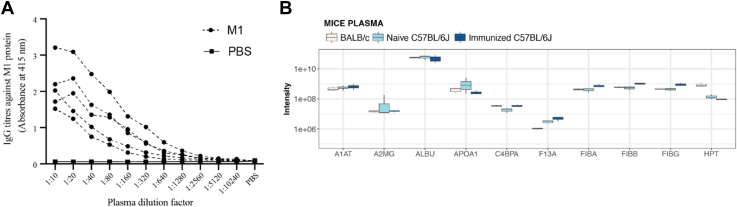
**Plasma proteome changes after immunization.** C57BL/6J mice were immunized with M1 or PBS and harvested after blood plasma was harvested after 6 weeks. *A*, ELISA analysis of IgG titers against M1 protein after serial dilution. *B*, the intensity distribution of proteins in plasma known to bind to the M1 in naive BALBC/c and mice C57BL/6J and in immunized C57BL/6J mice.

Once the mouse model system was established to investigate if an increase in anti-M1 IgG alters the composition, we immunized C57BL/J6 mice with the M1-protein. After 6 weeks, all animals demonstrated elevated titers against the M1-protein in plasma (Fig. 3*A* and supplemental Fig. S3). We first compared the plasma proteomes using DIA-MS from naïve and immunized C57BL/6J mice, to determine if immunization alters the abundance levels of the common binders in plasma, including haptoglobin, which was in previous crosslinking MS work shown to interacts with the C-terminal part of the M1 protein (18). In addition, we included BALB/c as an additional control to investigate if different mice strains have inherent differences in the plasma proteome composition. For most of the M1 protein interacting proteins, there were only minor changes in baseline plasma levels in response to immunization (supplemental Fig. S3). For *alpha-1 antitrypsin* (A1AT), C4BP, F13 and the three fibrinogen chains there was a slight increase in abundance level in the immunized animals (20.5%, 86.7%, 70.2% and 95.1%, respectively) whereas the levels of albumin and haptoglobin were slightly reduced (13.16% and 32.6%, respectively) (Fig. 3*B*).

### M1-Specific Antibodies and Impact on the M1 Protein Interaction Network

**Fig. 4:**
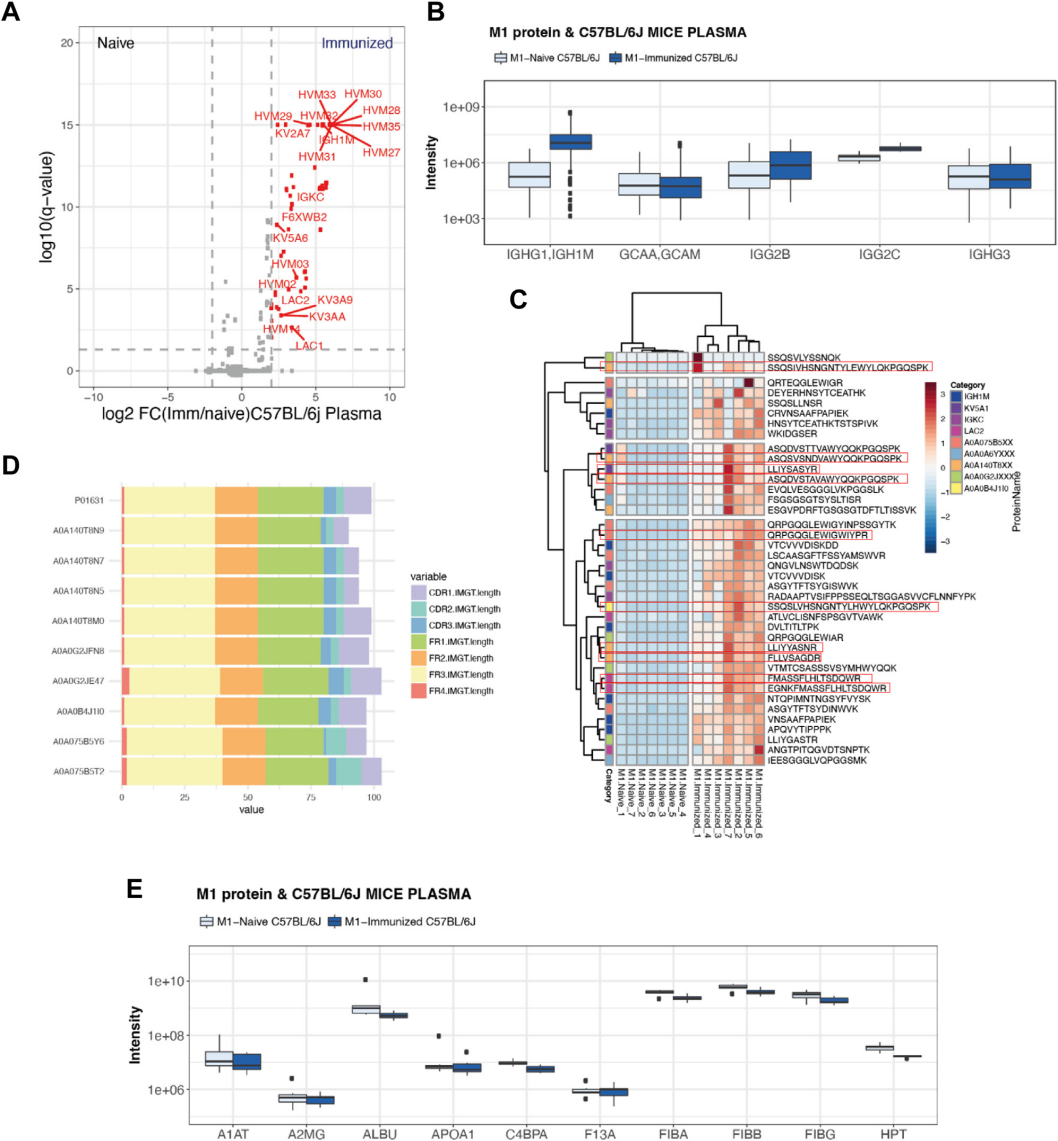
**Immunized mouse interactome against M1 protein.***A*, enriched M1 protein interaction in blood plasma from naïve C57BL/6J mice and immunized C57BL/6J mice. The volcano plots display significantly enriched mouse proteins comparing naïve and immunized C57BL/6J mice after correction of *p*-values with Benjamin-Hochberg on the y-axis and fold change on the x-axis. The dashed gray lines indicate log10 (*q*-value <0.05) and log2 fold change ≥2. The intensity for each protein is shown by the size of the dots. High-confident or TRUE interactions (*red dots*) while *grey dots* indicate contaminating proteins or FALSE interactions falling below the set thresholds (57). *B*, intensity distribution of IgG subclasses in plasma from naïve and immunized mice after AP-MS using IgG subclass specific and conserved proteotypic peptides as previously described (18). Acronyms: emm1 = M1 protein of *S. pyogenes (emm1).* IGH1M = IGHG1 membrane; GCAA = A alele and GCAM = membrane are IGG2A. *C*, HeatMap of the statistical significantly unique IgG peptides binding M1 proteins identified after AP-MS. Annotations rows show, the different groups of IgGs. *Boxes* represented the uniquely identified all min 7 detections. *D*, horizontal stacked bar chart figure showing the number of lengths for each variable region (V) of immunoglobulins G (IG) complementarity-determining regions (CDR) and framework regions (FR). *E*, distribution of mice blood plasma proteins known to bind M1 protein (18) after AP-MS analysis from plasma from naïve and immunized animals. Boxes indicate the median and interquartile ranges; whiskers indicate the range.

Next, we investigated if the anti-M1 antibodies generated after immunization altered the topology of the M1 protein interaction network by affinity-enriched plasma proteins from C57BL/J6 naïve and immunized mice followed by DIA-MS. In previous work, we have shown that the M1 interaction sites are saturated in pooled normal host plasma (15). In addition, we observed the interacting proteins in the flow-through indicating that the M1 protein is presented in sub-saturation levels. The AP-MS experiment from C57BL/J6 naïve and immunized mice generated 56 significant immunoglobulin chains with a fold-change of ≥ 4 (Fig. 4*A* and supplemental Fig. S3). Focusing on the sub-class specific proteotypic peptides of the conserved constant regions indicates that the increased binding of murine immunoglobulins to the M1 protein after immunization are connected to the subclasses of mouse IgGs, IGHG1/IGH1M, and to some degree IGG2C (Fig. 4*B* and supplemental Fig. S3).

To further characterize the affinity-enriched IgGs, we relied on previously established IgG repositories from IMGT and UniProt. Comparing the results from BALB/c and C57BL/J6, we found several peptide sequences that were shared among all experiments (supplemental Tables S3 and S4). We filtered the immunoglobulin peptide sequences by unique peptides to compare the most abundant peptide sequences through all experiments (supplemental Fig. S3). This analysis showed that several peptide sequences belong to IgG1 and were significantly enriched in the immunized animals, where the most predominant peptide sequence was VNSAAFPAPIEK. In the immunized animals, we identified 292 peptide sequences associated with the 56 enriched immunoglobulin chains from which 50 unique peptides were significant (Fig. 4, *C* and *D*). In the final step, we mapped all IgG peptides enriched in the AP-MS experiments from the immunized animals to immunoglobulin sequences found in UniProt/SwissProt and retrieved the full sequences. These sequences were blasted against the IMGT immunoglobulin database. The length of each variable region (V) of the IgG complementarity-determining regions (CDR) and framework regions (FR) is shown in Figure 4*D*. The retrieved sequences can only act as representative sequences, as we did not have access to the B cell receptor sequence repertoire from the mice included in this study. Nevertheless, we note that the homology for V and J regions among the 8 kappa variable regions was more than 99% (99.14 and 99.32%, respectively), indicating a possible antigen-specific polyclonal expansion against the same epitope (supplemental Tables S3 and S4).

The increase in M1 protein antigen-specific polyclonal antibodies did however not alter the levels of the binders shown in Figures 1 and 2. The volcano plot in Figure 4*A* shows that none of the binders were reduced significantly compared to the AP-MS results from naïve and immunized animals. Plotting the intensities of the binders revealed there was a slight non-significant reduction of these proteins in the immunized mice (Fig. 4*E* and supplemental Fig. S4). The most pronounced effect was observed for fibrinogen and haptoglobin. The levels of haptoglobin were reduced in the plasma samples from the same mice (Fig. 3*B*), which could explain the lower intensities after AP-MS. Fibrinogen, on the other hand, was increased in the immunized samples by 85.2% for FIBA, 88.7% for FIBB, and 112.2% for FIBG and then reduced by 35.3% (32.3% for FIBB, 36.7% for FIBA, and 36.8% for FIBG) in the AP-MS results, indicating that this interaction can to some degree be prevented by specific antibodies from immunized mice. In conclusion, these results show that the immunization results in high titers of IgG antibodies against M1 protein, but these antibodies only had a minor effect in preventing acquisition of mouse plasma proteins by M1 protein. This relative inability of anti-M1 specific antibodies to outcompete the plasma protein binding indicates that the polyclonal anti-M1 IgG primarily targets epitopes outside the protein binding interfaces.

## Conclusion

Many significant bacterial pathogens produce surface-exposed and secreted proteins that can form protein interaction networks with human host proteins to evade the immune system, acquire metabolites, and facilitate adherence. The same protein repertoire is also the target for antibodies from the adaptive immune system to neutralize relevant functions for virulence, and to promote bacterial clearance. This dichotomy creates a complex host-pathogen interplay as maintaining binding to host proteins and avoiding antibody binding is essential for bacterial pathogens to adhere and colonize their host. This is particularly important for species like *S. pyogenes* where pre-existing circulating antibodies against many antigens are highly prevalent in the human population (56). Here, we propose an experimental strategy to measure the inherent competition between antibodies and interacting plasma proteins using AP-MS under immune and non-immune conditions. As a starting point, we first compared the interaction network between M1 protein and human/murine plasma proteins. Although, there are measurable differences between the networks formed, the most abundant proteins such as albumin, fibrinogen, and IgG were common. Several of these proteins have in other studies previously been shown to interact with the M1 protein in human plasma (15). These interactions were determined using pooled human plasma with measurable antibody titers against the M1 protein, indicating that these interactions are formed in the presence of antibodies. As *S. pyogenes* is an obligate human pathogen, mice do not have anti-M1 antibodies. Immunizing mice with the M1 protein did not alter the composition of the M1 murine plasma interaction network despite the immunization generated high antibody titters against the M1 protein. These results show that the significant increase in antibody titers does not outcompete the protein interactions formed under naïve conditions, indicating that the binding interfaces are subdominant or that the high levels of plasma proteins can still be formed despite presence of competing antibody clones. The latter explanation, however, is less plausible as many of the human plasma proteins binding M1 proteins have affinities in the micromolar range (32). It can be expected that high-affinity antibodies typically with affinities in the nanomolar range would be able to outcompete protein interactions with cognate human proteins as the affinities associated with these interactions are typically substantially higher (22). Although the strategy proposed here only provides indirect evidence that the antibodies do not target the epitopes in the binding interfaces, this approach has the added value of determining the subclasses of the antigen-specific antibodies. Here, the proteotypic peptides mapping of conserved Fc-parts of the immunoglobulins were primarily associated with IgG1, indicating that IgG1 is the major immunoglobulin after immunization in animals in this animal model system. This approach would further benefit from the full amino acid sequence and landscape of clonal families to generate more in-depth information on the antigen-specific IgG clones.

### Ethical Considerations

The animal use protocol was approved by the local Malmö/Lund Institutional Animal Care and Use Committee (M115-13).

## Data Availability

The MS data have been deposited in the ProteomeXchange member repository MassIVE with the identifier: MSV000091506 (data access for reviewers: ftp://massive.ucsd.edu/MSV000091506/).

## Supplemental Data

This article contains supplemental data (18, 57).

## Conflict of interest

The authors declare that they have no known competing financial interests or personal relationships that could have appeared to influence the work reported in this paper.
