# Supplementary material for: Quantification of Adaptive Immune Responses Against Protein-Binding Interfaces in the Streptococcal M1 Protein
Source: Mol Cell Proteomics. 2024 Mar 23;23(5):100753. doi: 10.1016/j.mcpro.2024.100753 (PMC11059317; doi:10.1016/j.mcpro.2024.100753)
Supplement: Supplemental Tables S3 and S4 [file mmc3.docx]

**Supplementary Table 3. Hits uniquely identified all min 7 detections using the peptides sequences from the significant immunized mouse proteins (Supp fig 4B, figure 3C).** For all hits (n = 54) were retrieved the metainfo including sequences, and for each hit was retrieved at peptide level detection evidence from precursors, protein group context and protein group names to select IgG that were ‘uniquely identified (in the context of UniProt). Protein sequence was queried on <http://www.imgt.org/blast/> using the tblastn program and and manually transferred top hit accession to http://www.imgt.org/ligmdb/search.actionStep 2: Manually transfer top hit accession to <http://www.imgt.org/ligmdb/search.action>.


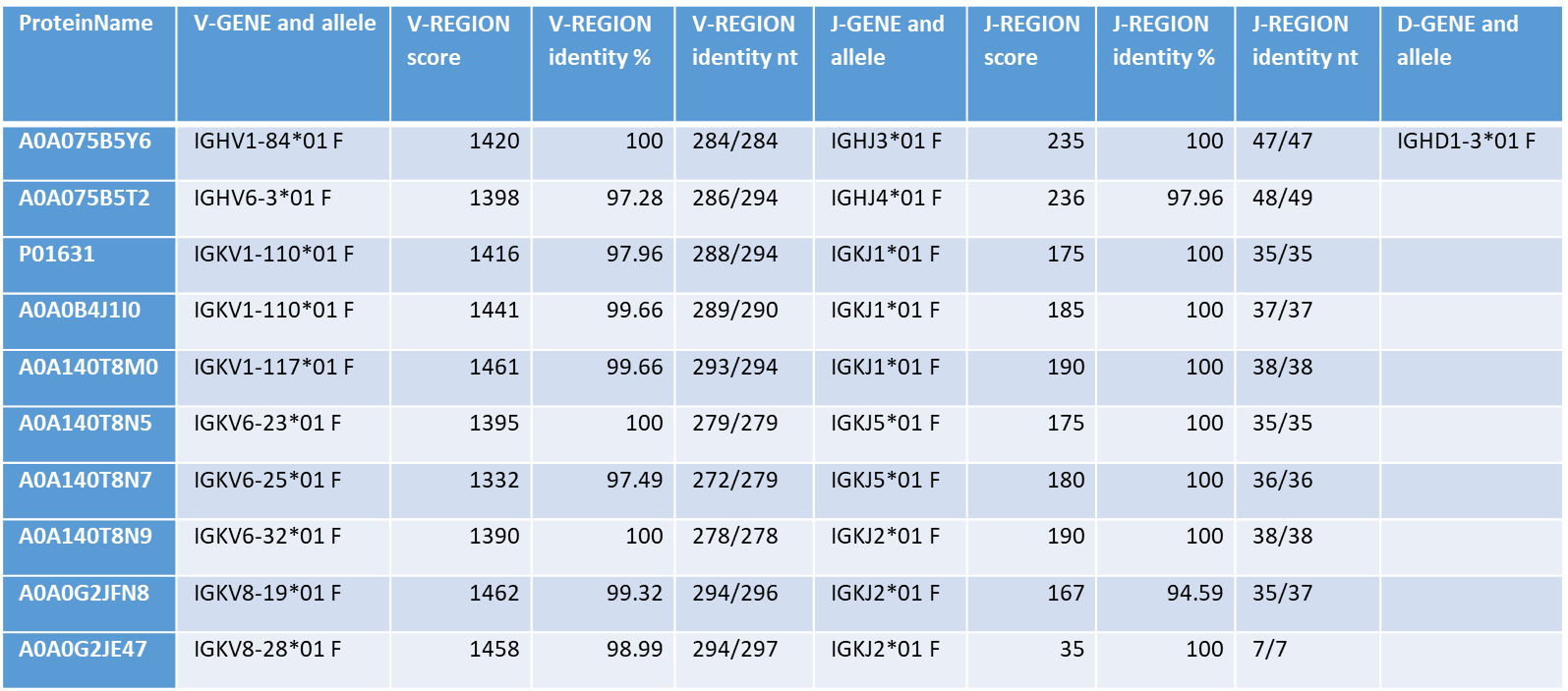


.

**Supplementary Table 4.** **VJD region of the 10 of the 12 IgGs uniquely identified all min 7 detections using the peptides sequences from the significant immunized mouse proteins (Supp fig 4B, figure 3C).** Protein sequence was queried on <http://www.imgt.org/blast/> using the tblastn program and and manually transferred top hit accession to http://www.imgt.org/ligmdb/search.actionStep 2: Manually transfer top hit accession to <http://www.imgt.org/ligmdb/search.action>

| **Protein accession** | **Gene accession** | **Protein** | **Sequence** | **V-D-J-REGION** | **V-J-REGION** | **V-REGION** | **FR1-IMGT** | **CDR1-IMGT** | **FR2-IMGT** | **CDR2-IMGT** | **FR3-IMGT** | **CDR3-IMGT** | **JUNCTION** | **J-REGION** | **FR4-IMGT** |
| --- | --- | --- | --- | --- | --- | --- | --- | --- | --- | --- | --- | --- | --- | --- | --- |
| **A0A075B5Y6** | AF045503_ (A0A075B5Y6) | 1/tr\|A0A075B5Y6\|A0A075B5Y6_MOUSE | MGWSWIFLFLLSGTAGVHSQVQLQQSGPELVKPGASVKLSCKASGYTFTSYDINWVKQRPGQGLEWIGWIYPRDGSTKYNEKFKGKATLTVDTSSSTAYMELHSLTSEDSAVYFCAR | QLQQSGPELVKPGASVKISCKASGYTFTDYYINWVKQRPGQGLEWIGWIYPGSGNTKYNEKFKGKATLTVDTSSSTAYMQLSSLTSEDSAVYFCARSDLTWFAYWGQGTLVTVSA |  | QLQQSGPELVKPGASVKISCKASGYTFTDYYINWVKQRPGQGLEWIGWIYPGSGNTKYNEKFKGKATLTVDTSSSTAYMQLSSLTSEDSAVYFCAR | QLQQSGPELVKPGASVKISCKAS | GYTFTDYY | INWVKQRPGQGLEWIGW | IYPGSGNT | KYNEKFKGKATLTVDTSSSTAYMQLSSLTSEDSAVYFC | ARSDLTWFAY | CARSDLTWFAYW | WFAYWGQGTLVTVSA | WGQGTLVTVSA |
| **A0A075B5T2** | BC031470_ (A0A075B5T2) | 1/tr\|A0A075B5T2\|A0A075B5T2_MOUSE | MDLRLSCAFIIVLLKGVQSEVKLEESGGGLVQPGGSMKLSCVASGFTFSNYWMNWVRQSPEKGLEWVAQIRLKSDNYATHYAESVKGRFTISRDDSKSSVYLQMNNLRAEDTGIYYCT | EVKLEESGGGLVQPGGSMKLSCVASGFTFSNSWMNWVRQSPEKGLEWVAQIRLRSDNYATHYAESVKGRFTISRDDSKSRLYLQMSSLRAEDTGIYYCTNAMDYWGQGTSVTVSS |  | EVKLEESGGGLVQPGGSMKLSCVASGFTFSNSWMNWVRQSPEKGLEWVAQIRLRSDNYATHYAESVKGRFTISRDDSKSRLYLQMSSLRAEDTGIYYCT | EVKLEESGGGLVQPGGSMKLSCVAS | GFTFSNSW | MNWVRQSPEKGLEWVAQ | IRLRSDNYAT | HYAESVKGRFTISRDDSKSRLYLQMSSLRAEDTGIYYC | TNAMDY | CTNAMDYW | AMDYWGQGTSVTVSS | WGQGTSVTVSS |
| **P01631** | X56623_ (P01631) | 1/sp\|P01631\|KV2A7_MOUSE | DVVMTQTPLSLPVSLGDQASISCRSSQSLVHSNGNTYLNWYLQKAGQSPKLLIYKVSNRFSGVPDRFSGSGSGTDFTLKISRVEAEDLGIYFCSQTTHVPPTFGGGTKLEIKR |  | DVVMTQTPLSLPVSLGDQASISCRSSQSLVHSNGNTYLNWYLQKAGQSPKLLIYKVSNRFSGVPDRFSGSGSGTDFTLKISRVEAEDLGIYFCSQTTHVPPTFGGGTKLEIK | DVVMTQTPLSLPVSLGDQASISCRSSQSLVHSNGNTYLNWYLQKAGQSPKLLIYKVSNRFSGVPDRFSGSGSGTDFTLKISRVEAEDLGIYFCSQTTHVP | DVVMTQTPLSLPVSLGDQASISCRSS | QSLVHSNGNTY | LNWYLQKAGQSPKLLIY | KVS | NRFSGVPDRFSGSGSGTDFTLKISRVEAEDLGIYFC | SQTTHVPPT | CSQTTHVPPTF | TFGGGTKLEIK | FGGGTKLEIK |
| **A0A0B4J1I0** | M32384_ (A0A0B4J1I0) | 1/tr\|A0A0B4J1I0\|A0A0B4J1I0_MOUSE | MKLPVRLLVLMFWIPASSSDVVMTQTPLSLPVSLGDQASISCRSSQSLVHSNGNTYLHWYLQKPGQSPKLLIYKVSNRFSGVPDRFSGSGSGTDFTLKISRVEAEDLGVYFCSQSTHVP |  | VMTQTPLSLPVSLGDQASISCRSSQSLVHSNGNTYLHWYLQKPGQSPKLLIYKVSNRFSGVPDRFSGSGSGTDFTLKISRVEAEDLGVYFCSQSTHVPWTFGGGTKLEIK | VMTQTPLSLPVSLGDQASISCRSSQSLVHSNGNTYLHWYLQKPGQSPKLLIYKVSNRFSGVPDRFSGSGSGTDFTLKISRVEAEDLGVYFCSQSTHV | VMTQTPLSLPVSLGDQASISCRSS | QSLVHSNGNTY | LHWYLQKPGQSPKLLIY | KVS | NRFSGVPDRFSGSGSGTDFTLKISRVEAEDLGVYFC | SQSTHVPWT | CSQSTHVPWTF | WTFGGGTKLEIK | FGGGTKLEIK |
| **A0A140T8M0** | X87231_ (A0A140T8M0) | 1/tr\|A0A140T8M0\|A0A140T8M0_MOUSE | MKLPVRLLVLMFWIPASSSDVLMTQTPLSLPVSLGDQASISCRSSQSIVHSNGNTYLEWYLQKPGQSPKLLIYKVSNRFSGVPDRFSGSGSGTDFTLKISRVEAEDLGVYYCFQGSHVP |  | DVLMTQTPLSLPVSLGDQASISCRSSQSIVHSNGNTYLEWYLQKPGQSPKLLIYKVSNRFSGVPDRFSGSGSGTDFTLKISRVEAEDLGVYYCFQGSHVPWTFGGGTKLEIK | DVLMTQTPLSLPVSLGDQASISCRSSQSIVHSNGNTYLEWYLQKPGQSPKLLIYKVSNRFSGVPDRFSGSGSGTDFTLKISRVEAEDLGVYYCFQGSHV | DVLMTQTPLSLPVSLGDQASISCRSS | QSIVHSNGNTY | LEWYLQKPGQSPKLLIY | KVS | NRFSGVPDRFSGSGSGTDFTLKISRVEAEDLGVYYC | FQGSHVPWT | CFQGSHVPWTF | WTFGGGTKLEIK | FGGGTKLEIK |
| **A0A140T8N5** | DQ220793_ (A0A140T8N5) | 1/tr\|A0A140T8N5\|A0A140T8N5_MOUSE | METHSQVFVYMLLWLSGVEGDIVMTQSHKFMSTSVGDRVSITCKASQDVGTAVAWYQQKPGQSPKLLIYWASTRHTGVPDRFTGSGSGTDFTLTISNVQSEDLADYFCQQYSSYP |  | DIVMTQSHKFMSTSVGDRVSITCKASQDVGTAVAWYQQKPGQSPKLLIYWASTRHTGVPDRFTGSGSGTDFTLTISNVQSEDLADYFCQQYSSYPLTFGAGTKLELK | DIVMTQSHKFMSTSVGDRVSITCKASQDVGTAVAWYQQKPGQSPKLLIYWASTRHTGVPDRFTGSGSGTDFTLTISNVQSEDLADYFCQQYSSYP | DIVMTQSHKFMSTSVGDRVSITCKAS | QDVGTA | VAWYQQKPGQSPKLLIY | WAS | TRHTGVPDRFTGSGSGTDFTLTISNVQSEDLADYFC | QQYSSYPLT | CQQYSSYPLTF | TFGAGTKLELK | FGAGTKLELK |
| **A0A140T8N7** | X05877_ (A0A140T8N7) | 1/tr\|A0A140T8N7\|A0A140T8N7_MOUSE | MESQIQAFVFVFLWLSGVDGDIVMTQSHKFMSTSVGDRVSITCKASQDVSTAVAWYQQKPGQSPKLLIYWASTRHTGVPDRFTGSGSGTDYTLTISSVQAEDLALYYCQQHYSTP |  | DIVMTQSHRFMSTSVGDRVSITCKASQDVTTAVSWYQQKPGQSPKLLIFWASTRHTGVPDRFTGSGSGTDYTLTISSVQAEDLALYYCQQHYSTPLTFGAGTKLELK | DIVMTQSHRFMSTSVGDRVSITCKASQDVTTAVSWYQQKPGQSPKLLIFWASTRHTGVPDRFTGSGSGTDYTLTISSVQAEDLALYYCQQHYSTP | DIVMTQSHRFMSTSVGDRVSITCKAS | QDVTTA | VSWYQQKPGQSPKLLIF | WAS | TRHTGVPDRFTGSGSGTDYTLTISSVQAEDLALYYC | QQHYSTPLT | CQQHYSTPLTF | TFGAGTKLELK | FGAGTKLELK |
| **A0A140T8N9** | AF045513_ (A0A140T8N9) | 1/tr\|A0A140T8N9\|A0A140T8N9_MOUSE | MKSQTQVFVFLLLCVSGAHGSIVMTQTPKFLLVSAGDRVTITCKASQSVSNDVAWYQQKPGQSPKLLIYYASNRYTGVPDRFTGSGYGTDFTFTISTVQAEDLAVYFCQQDYSSP |  | IVMTQTPKFLLVSAGDRVTITCKASQSVSNDVAWYQQKPGQSPKLLIYYASNRYTGVPDRFTGSGYGTDFTFTISTVQAEDLAVYFCQQDYSSPYMYTFGGGTKLEIK | IVMTQTPKFLLVSAGDRVTITCKASQSVSNDVAWYQQKPGQSPKLLIYYASNRYTGVPDRFTGSGYGTDFTFTISTVQAEDLAVYFCQQDYSS | IVMTQTPKFLLVSAGDRVTITCKAS | QSVSND | VAWYQQKPGQSPKLLIY | YAS | NRYTGVPDRFTGSGYGTDFTFTISTVQAEDLAVYFC | QQDYSSPYMYT | CQQDYSSPYMYTF | YTFGGGTKLEIK | FGGGTKLEIK |
| **A0A0G2JFN8** | FN686799_ (A0A0G2JFN8) | 1/tr\|A0A0G2JFN8\|A0A0G2JFN8_MOUSE | MESQTQVLMSLLFWVSGTCGDIVMTQSPSSLTVTAGEKVTMSCKSSQSLLNSGNQKNYLTWYQQKPGQPPKLLIYWASTRESGVPDRFTGSGSGTDFTLTISSVQAEDLAVYYCQNDYSYP |  | IVMTQSPSSLTVTAGEKVTMSCKSSQSLLNSGNQKNYLTWHQQKPGQPPKLLIYWASTRESGVPDRFTGSGSGTDFTLTISSVQAEDLAVYYCQNDYSYYTFGGGTKLEIR | IVMTQSPSSLTVTAGEKVTMSCKSSQSLLNSGNQKNYLTWHQQKPGQPPKLLIYWASTRESGVPDRFTGSGSGTDFTLTISSVQAEDLAVYYCQNDYSY | IVMTQSPSSLTVTAGEKVTMSCKSS | QSLLNSGNQKNY | LTWHQQKPGQPPKLLIY | WAS | TRESGVPDRFTGSGSGTDFTLTISSVQAEDLAVYYC | QNDYSYYT | CQNDYSYYTF | YTFGGGTKLEIR | FGGGTKLEIR |
| **A0A0G2JE47** | X59816_ (A0A0G2JE47) | 1/tr\|A0A0G2JE47\|A0A0G2JE47_MOUSE | MESQTQVLISLLFWVSGTCGDIVMTQSPSSLSVSAGEKVTMSCKSSQSLLNSGNQKNYLAWYQQKPGQPPKLLIYGASTRESGVPDRFTGSGSGTDFTLTISSVQAEDLAVYYCQNDHSYP |  | DIVMTQSPSSLTVSAGEKVTMSCKSSQSLFNSGTQKNYLAWYQQKPGQPPKLLIYGASTRESGVPDRFTGSGSGTDFTLTISSVQAEDLAVYYCQNDHSYPYT | DIVMTQSPSSLTVSAGEKVTMSCKSSQSLFNSGTQKNYLAWYQQKPGQPPKLLIYGASTRESGVPDRFTGSGSGTDFTLTISSVQAEDLAVYYCQNDHSY | DIVMTQSPSSLTVSAGEKVTMSCKSS | QSLFNSGTQKNY | LAWYQQKPGQPPKLLIY | GAS | TRESGVPDRFTGSGSGTDFTLTISSVQAEDLAVYYC | QNDHSYPYT |  | YT |  |
| **P01844** | X58414_ (P01844) | 1/sp\|P01844\|LAC2_MOUSE | QPKSTPTLTVFPPSSEELKENKATLVCLISNFSPSGVTVAWKANGTPITQGVDTSNPTKEGNKFMASSFLHLTSDQWRSHNSFTCQVTHEGDTVEKSLSPAECL |  |  |  |  |  |  |  |  |  |  |  |  |
| **A0A075B5Y4** |  | 1/tr\|A0A075B5Y4\|A0A075B5Y4_MOUSE | MEWIWIFLFILSGTAGVQSQVQLQQSGAELARPGASVKLSCKASGYTFTSYGISWVKQRTGQGLEWIGEIYPRSGNTYYNEKFKGKATLTADKSSSTAYMELRSLTSEDSAVYFCAR |  |  |  |  |  |  |  |  |  |  |  |  |
