# Supplementary material for: Quantification of Adaptive Immune Responses Against Protein-Binding Interfaces in the Streptococcal M1 Protein
Source: Mol Cell Proteomics. 2024 Mar 23;23(5):100753. doi: 10.1016/j.mcpro.2024.100753 (PMC11059317; doi:10.1016/j.mcpro.2024.100753)
Supplement: Supplemental Data [file mmc4.docx]

**Supplementary Material**

**for**

**Quantification of adaptive immune responses against protein binding interfaces in the streptococcal M1 protein using AP-MS**

Torres-Sangiao E^1,2,3,#^, Happonen L^1^, Heusel M^1,4^, Palm, F^1^, Gueto Tettay C^1^, Malmström L^1^, Shannon O^1,5^ and Malmström J^1,#^

^1^Lund University, Faculty of Medicine, Department of Clinical Sciences Lund, Division of Infection Medicine. BMC D13, SE-22184, Lund, Sweden.

^2^University Hospital Complex – Research Institute of Santiago de Compostela (CHUS-IDIS), Microbiology, *Escherichia coli* group. Santiago de Compostela, Spain.

^3^CHUS-IDIS, Clinical Microbiology Lab. Santiago de Compostela, Spain.

^4^ Evosep ApS, Buchwaldsgade 35, Odense, Denmark

^5^ Section for Oral Biology and Pathology, Faculty of Odontology, Malmö University, 20506 Malmö, Sweden.

# Corresponding author: Johan Malmström: [johan.malmstrom@med.lu.se](mailto:johan.malmstrom@med.lu.se)

Lund University, Faculty of Medicine, Department of Clinical Sciences Lund, Division of Infection Medicine. BMC D13, SE-22184, Lund, Sweden.

# Corresponding author: Eva Torres-Sangiao: [eva.torres.sangiao@gmail.com](mailto:eva.torres.sangiao@gmail.com)

Health Research Institute of Santiago de Compostela (IDIS), *Escherichia coli* group. University Hospital Complex of Santiago de Compostela, Clinical Microbiology Lab. 15706 Santiago de Compostela, Spain.

**Supplementary Figure 1:**

**Mouse and human plasma proteins were affinity enriched in nine biological replicates using tagged M1 protein and the enriched proteins were identified and quantified using mass spectrometry (AP-MS)**. A - B) Intensity distribution in sfGFP (Ctrl) and M1 protein (M1) in the AP-MS experiments of human plasma proteins known to bind to the M1 protein^1^, alpha-2-macroblobulin (A2MG) and mouse A1AT calculated as mean of the six domains (A1AT*1-6*) for A) human plasma proteins and B) mouse BALB/c plasma proteins. See Table S1 for more information and protein acronyms.

**Supplementary Figure 2:**

A) A tagged M1 protein was used to enrich mouse and human plasma proteins from a (50% vol/vol) mixture of human and BALB/c mouse plasma followed by mass spectrometry analysis. The bar chart distribution displays the abundance of significantly enriched mouse or human specific proteins binding M1 protein from figure 1, without immunoglobulins fragments. Protein abundance is expressed as log2 fold change. Fold change has been expressed as ratio plasmas samples with M1 protein (M1) / plasma samples with sfGFP or negative control (Ctrl).

B) Distribution of plasma proteins known to bind M1 protein^1^ and alpha-2-macroblobulin from competition experiments (mouse and human plasma in 50% vol/vol mixture), considering the global analysis or each individual human *vs* BALB/c mouse plasma. Mouse A1AT value has been calculated as mean of the six domains (A1AT*1-6*). Acronyms: emm1=M1 protein of *S. pyogenes (emm1).*

C) HeatMap for the hierarchical clustering of the statistical significantly peptides binding M1 protein from AP competition experiments (mixed human and mouse blood plasma). High statistical significances were displayed according to *p*-value < 0.01 and a log2 fold change > 2. Annotations rows show, the different groups of mammalian blood sample and M1 protein (emm1) from *S. pyogenes*, the different groups of proteins classified as fibrinogen (FIB), immunoglobulins (IGs), M1 protein of *S. pyogenes* (emm1), and other proteins group, and last annotation showing the common and unique peptides. For more information see supplementary table S3.

D) HeatMap for the hierarchical clustering of the statistical significantly peptides binding M1 protein from surface adsorption experiments. High statistical significances were displayed according to *p*-value < 0.01 and large magnitude fold changes as log2 fold change > 2. Annotations rows show, the different groups of mammalian blood sample and M1 protein (emm1) from *S. pyogenes*, the different groups of proteins classified as fibrinogen (FIB), immunoglobulins (IGs), M1 protein of *S. pyogenes* (emm1), and other proteins group, and last annotation showing the common and unique peptides. For more information see supplementary table S3

.

**Supplementary Figure 3:**

**A)** Differential abundance of M1 protein`s interactions and blood plasma proteins of immunized C57BL/6J mice, compared to BALB/c negative control. Volcano Plots display a statistical significances according to Benjamini-Hochberg adjusted *q*-value < 0.05 (y-axis), and large magnitude fold changes (FC) (x-axis). The dashed grey-line shows log10 (*q*-value <0.05) and log2 fold change > 2. The absolute intensity is shown by size dots. High-confident or TRUE interactions (red dots) while grey dots indicate contaminating proteins or FALSE interactions falling below the set thresholds^2^, aforementioned. Multiple testing corrections were performed by the Benjamini-Hochberg method. Fold change (FC) has been expressed as ratio plasmas samples with M1 protein (M1) / plasma samples with sfGFP or negative control (Ctrl); and immunized (Imm) *versus* naïve C57BL/6J plasma samples, both with M1 protein. Red Dot not labed belong to fragments of Ig heavy or kappa chain variable. emm1=M1 protein of *S. pyogenes (emm1).*

**B)** Heatmap for the hierarchical clustering of significantly enriched blood plasma proteins comparing naïve and immunized C57BL/6J mice samples. High significances were selected according to *p*-value < 0.05 and large magnitude fold changes as log2 fold change > 2. Columns represent samples and rows represent the blood plasma proteins below the pre-established set. The scale bar indicates *z*-scores of values with highly abundance depicted in dark red low abundance proteins depicted in dark blue. The keratins were removed from all heatmaps. d)

**C**) Distribution of IgGs across BALC/c and C57BL/6J mice blood plasma samples, using using IgG subclass specific and conserved proteotypic peptides. Boxes indicate the median and interquartile range; whiskers indicate the range. NOTE: IGH1M= IGHG1 membrane; GCAA= A alele and GCAM=membrane are IGG2A.

**D**) The M1 protein –mice plasma interactome. Bubbles size = fold change. Width line = log normalized mean. Transparency line = number of peptides identified per protein. The interactome views were generated using Cytoscape and modified in Adobe Illustrator.

**Supplementary Figure 4:**

**A) HeatMap for the hierarchical clustering of unique proteotypic IgGs peptides from different types of profile mice plasma and experiments.** Columns represent samples. The scale bar indicates *z*-scores of log and values, respectively, with highly abundance /interactions depicted in dark red low abundance / interactions proteins depicted in dark blue (*see legend*). The clustering shows a clear separation between healthy pooled plasma (BALB/c mouse) and immunized and non-immunized (C57BL/6J), as well as plasma samples and AP samples. Annotations show, Mouse.spp (annotation column) the two different species of mouse used for the AP experiments, and the two different procedures, cell lysis or plasma and AP. Category (annotation row) the different subclasses of IgGs defined for Mouse; Enrichment threshold (annotations rows) show the peptide which intensity were below the pre-established set thresholds (*q*-value < 0.05 and fold change > 2. Fold change has been expressed as ratio immunized / naïve C57BL/6J plasma samples (enrichment_ ImmNaive), BALB/c plasma / C57BL/6J plasma samples (enrichment _PlasmaNaive), and AP samples BALB/c plasma / C57BL/6J plasma samples (enrichment _M1_PlasmaNaive), immunized / non immunized C57BL/6J plasma samples (M1_enrichment_ ImmNaive) and BALB/c plasma / negatice control BALB/c plasma samples (enrichment _M1_Plasma). The clustering shows a clear separation according to the different species of mouse.

**B - E) Visualization of the alignment of the 12 sequences peptides assembled (using IMGT database).** Target sequence on the top, annotated variable domains and covered region for each peptide (rectangles). The green segments represent unique peptides for a particular protein while the grey one match 2 or more sequences.

**F) Supplementary Table. Hits uniquely identified all min 7 detections using the peptides sequences from the significant immunized mouse proteins (Supp fig 4B, figure 3C).** For all hits (n = 54) were retrieved the metainfo including sequences, and for each hit was retrieved at peptide level detection evidence from precursors, protein group context and protein group names to select IgG that were ‘uniquely identified (in the context of UniProt). Protein sequence was queried on <http://www.imgt.org/blast/> using the tblastn program and and manually transferred top hit accession to http://www.imgt.org/ligmdb/search.actionStep 2: Manually transfer top hit accession to <http://www.imgt.org/ligmdb/search.action>.

**G) Table.** **VJD region of the 10 of the 12 IgGs uniquely identified all min 7 detections using the peptides sequences from the significant immunized mouse proteins (Supp fig 4B, figure 3C).** Protein sequence was queried on <http://www.imgt.org/blast/> using the tblastn program and and manually transferred top hit accession to http://www.imgt.org/ligmdb/search.actionStep 2: Manually transfer top hit accession to <http://www.imgt.org/ligmdb/search.action>.

**Supplementary Figure 5:**

**A) Venn Diagram for AP-MS mouse** experiments showing the general overlap with all proteins that have p < 0.05, fold change (FC) > 1.5 and >= 2 unique peptides (pep). We individually show the overlap for proteins identified by more than 1 peptide and identified by more than 2 peptides. Differential abundance of interactions comparing immunized (IMM) versus naïve mice and each C57BL/6J and BALC/c AP-MS experiments versus negative control. We individually show the overlap for proteins identified by more than 1 peptide and identified by more than 2 peptides, as well as FC > 1.5 or between 1.5 – 2**. B) Pearson correlation multiplot** of the ratios of negative control versus samples for all AP-MS experiments using 25 microliters of each mouse plasma.

References

1. Hauri, S. *et al.* Rapid determination of quaternary protein structures in complex biological samples. *Nat Commun* **10**, 192 (2019).

2. Collins, B.C. *et al.* Quantifying protein interaction dynamics by SWATH mass spectrometry: application to the 14-3-3 system. *Nat Methods* **10**, 1246-1253 (2013).
