# Supplementary material for: Quantification of Adaptive Immune Responses Against Protein-Binding Interfaces in the Streptococcal M1 Protein
Source: Mol Cell Proteomics. 2024 Mar 23;23(5):100753. doi: 10.1016/j.mcpro.2024.100753 (PMC11059317; doi:10.1016/j.mcpro.2024.100753)

Supplement Figure 1: Torres-Sangiao *et al*

A

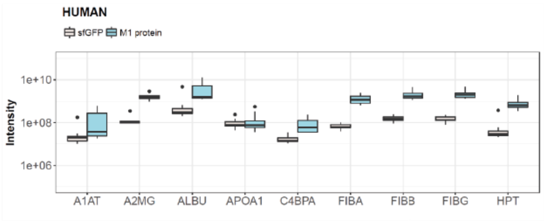

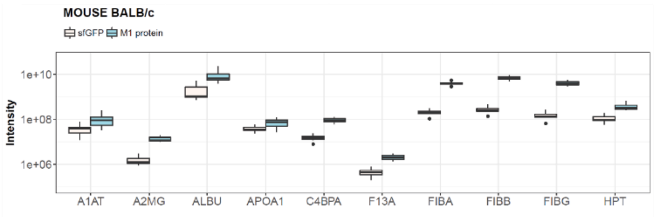

Supplement Figure 2: Torres-Sangiao *et al*

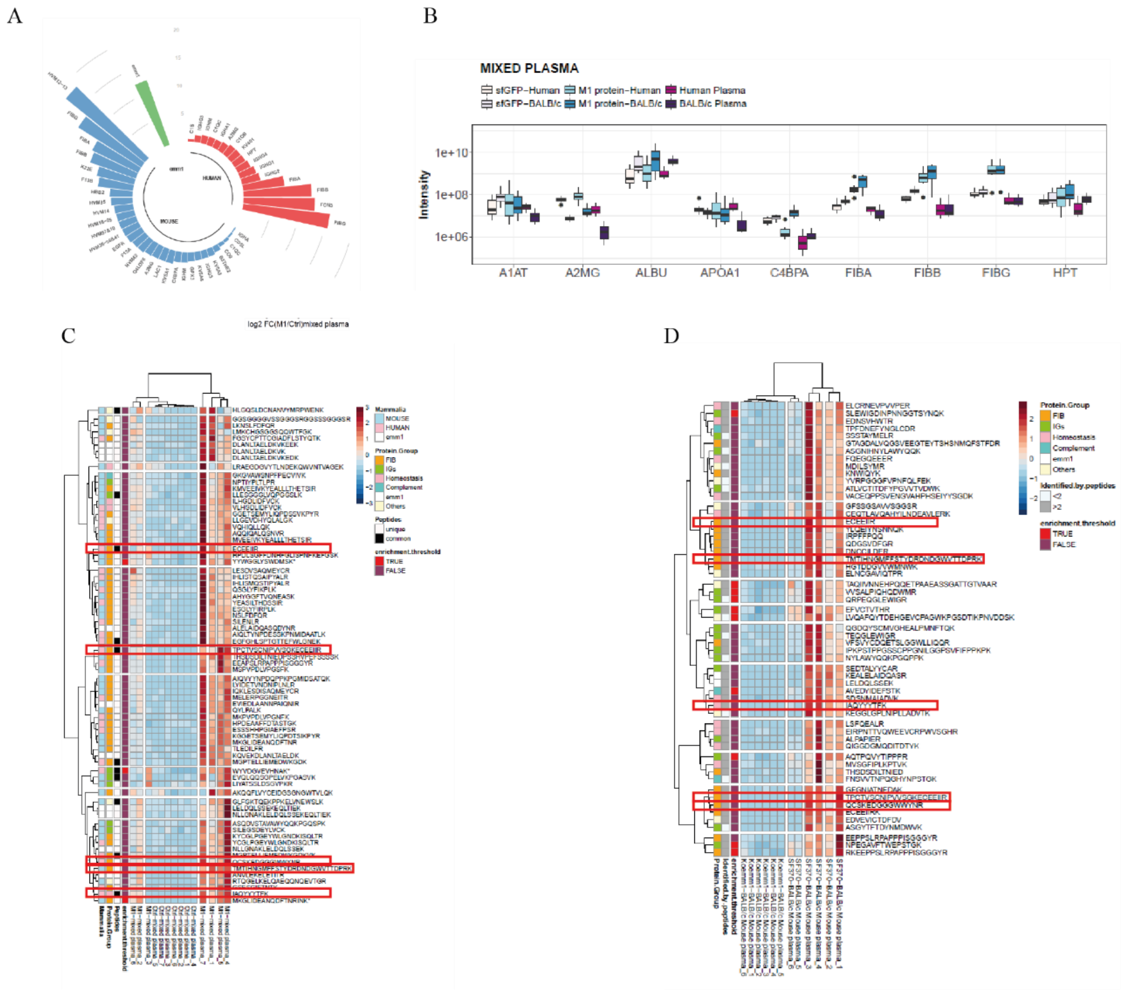

.

Supplement Figure 3: Torres-Sangiao *et al*

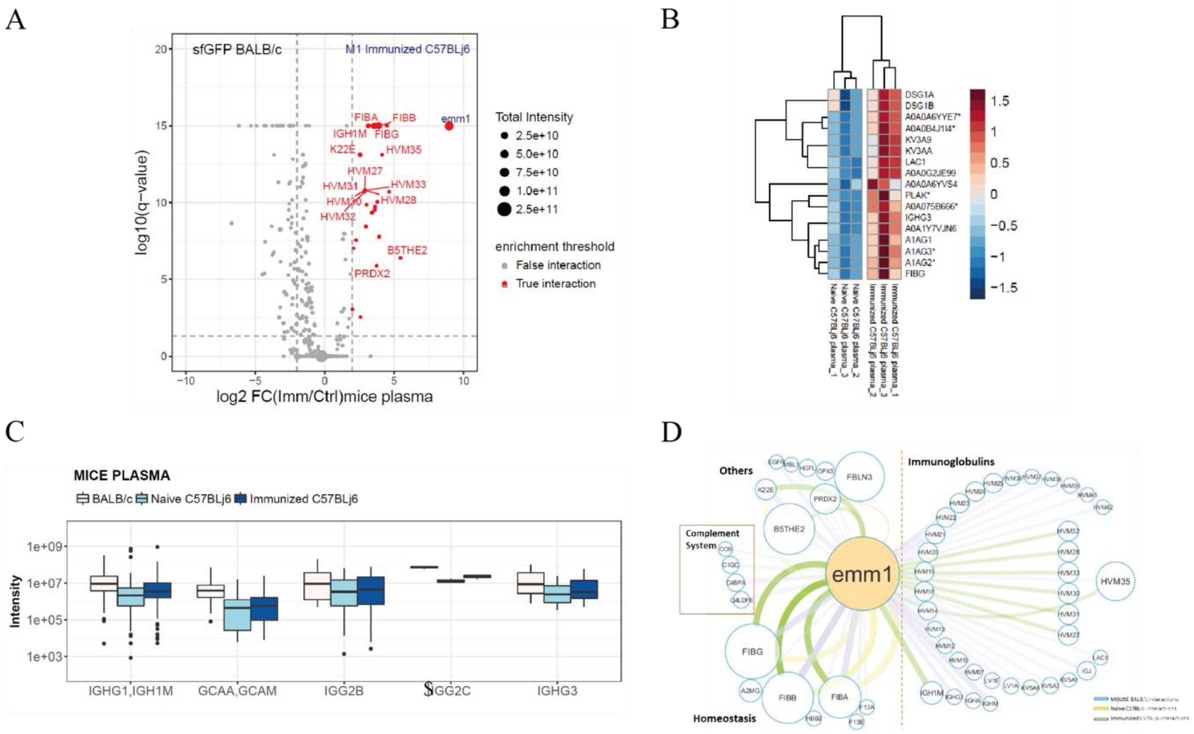

Supplement Figure 4: Torres-Sangiao *et al*

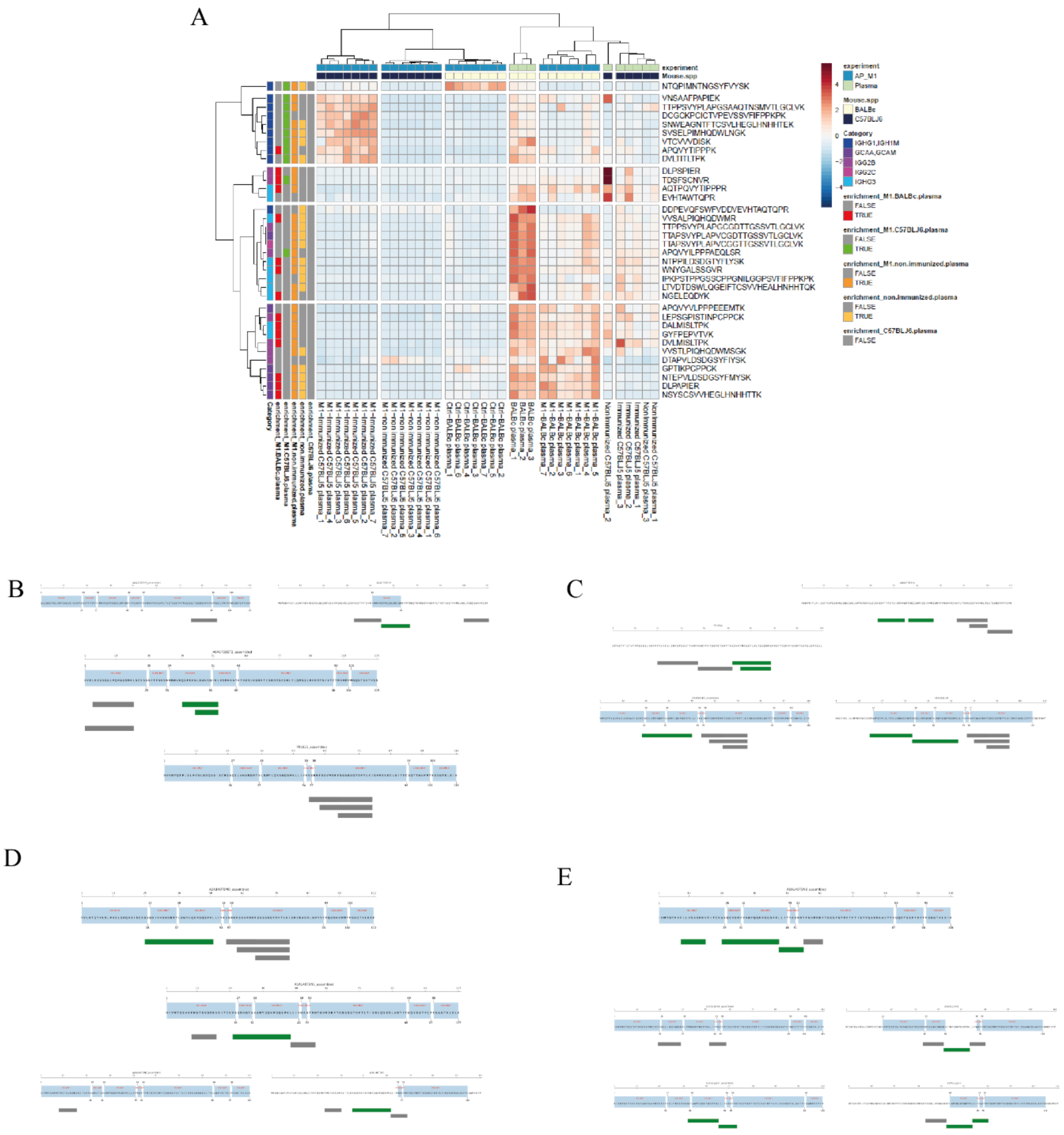

Supplement Figure 5: Torres-Sangiao *et al*

A

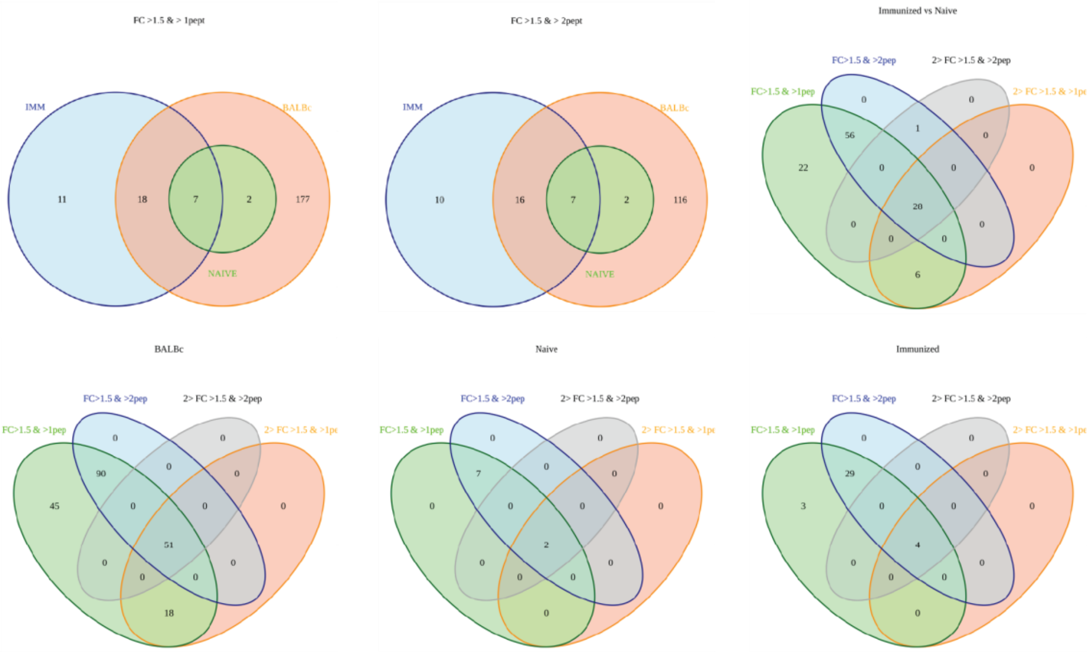

B

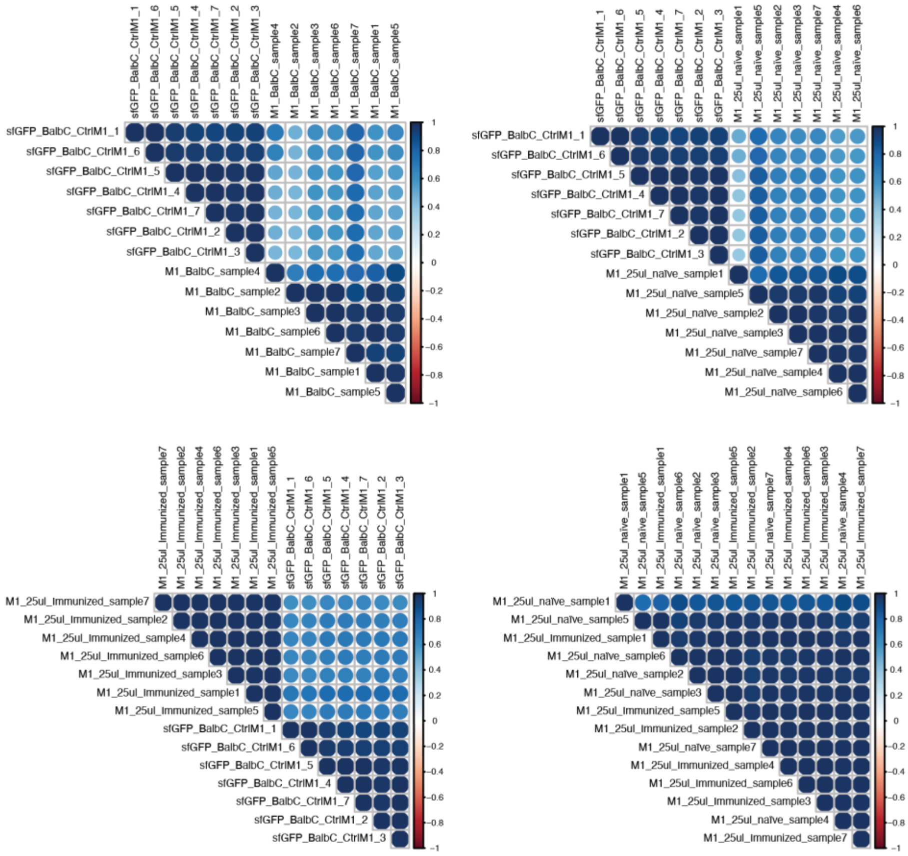

Supplement: Supplemental Figures S1–S5 [file mmc5.pdf]
